# Genetic-epigenetic interaction in lupus

**DOI:** 10.1186/ar3943

**Published:** 2012-09-27

**Authors:** AH Sawalha

**Affiliations:** 1 University of Michigan, Ann Arbor, MI, USA

## 

Systemic lupus erythematosus is a genetically complex autoimmune disease. A large body of evidence suggests an important role for epigenetic variation, particularly DNA methylation changes, in the pathogenesis of lupus. We recently performed a comprehensive evaluation of the DNA methylome in lupus T cells and identified a number of differentially methylated loci that can contribute to the pathogenesis of the disease. By analyzing the interaction between genetic risk, T-cell DNA demethylation, and the SLEDAI scores, we demonstrated that the (genetic risk)/(T-cell DNA methylation) ratio increased directly with disease activity in lupus patients. Furthermore, men with lupus require a higher genetic risk and/or lower T-cell DNA methylation levels to achieve a lupus flare of equal severity to women, suggesting genetic-epigenetic interaction in explaining the sex bias in lupus. We have also established the genetic region containing methyl-CpG-binding protein 2 (*MECP2*) as a lupus susceptibility locus. MeCp-2 is a key transcription factor critically involved in regulating the expression of methylation-sensitive genes, and directly recruits DNA methyltransferase 1 (DNMT1). Indeed, recent data from our group demonstrate that the lupus-associated variants in *MECP2 *induce DNA methylation changes in key inflammatory genes. Our data suggest that efforts to study genetic-epigenetic interactions in lupus will further our understanding of the disease pathogenesis and might help to explain, at least in part, the missing heritability in lupus.

